# Epithelioid Angiosarcoma Arising from a Huge Leiomyoma: A Case Report and a Literature Review

**DOI:** 10.1155/2018/7591769

**Published:** 2018-06-04

**Authors:** Takeya Hara, Ai Miyoshi, Yuji Kamei, Nao Wakui, Akiko Fujishiro, Serika Kanao, Hirokazu Naoi, Hirofumi Otsuka, Takeshi Yokoi

**Affiliations:** Department of Obstetrics and Gynecology, Kaizuka City Hospital, Osaka, Japan

## Abstract

Uterine mesenchymal tumors other than leiomyosarcoma, carcinosarcoma, and endometrial stromal sarcomas are extremely uncommon. We describe a case of epithelioid angiosarcoma of the uterus and review previous literature on such rare tumors. A 48-year-old woman presented with a 1-year history of abdominal fullness and 10kg weight loss. Pelvic magnetic resonance imaging (MRI) revealed a huge (30×18cm) uterus accompanied by degeneration and necrosis. She underwent supracervical hysterectomy and right salpingo-oophorectomy. We postoperatively diagnosed the mass as an epithelioid angiosarcoma arising from a leiomyoma. Vasodilatation was observed within the range of 2 cm × several mm in the leiomyoma, and proliferation of atypical cells was observed covering the surface of the luminal side. The tumor showed a partly fine vascular structure and was associated with obvious nuclear atypia and mitotic figures. She received 6 courses of adjuvant chemotherapy with paclitaxel, epirubicin, and carboplatin, and there have been no signs of recurrence for 10 months.

## 1. Introduction

Angiosarcoma is defined as a tumor of the endothelial cells presenting in blood vessels. Microscopically, the tumor is composed of anastomosing vascular tubes, having endothelial cells in larger numbers than needed to line the vessels [1]. Angiosarcoma accounts for less than 1% of all soft tissue sarcomas. It is an aggressive and malignant soft tissue neoplasm [2]. Although angiosarcoma can arise in any region of the body, most occur in the skin or superficial soft tissue in the elderly [3]. Uterine mesenchymal tumors other than leiomyosarcoma, carcinosarcoma, and endometrial stromal sarcomas are uncommon. Primary uterine epithelioid angiosarcoma is an extremely rare malignant tumor with a poor prognosis. We report here a new case of epithelioid angiosarcoma arising in a leiomyoma of the uterus and we include a literature review concerning the previous similar cases in the past 50 years.

## 2. Case Presentation

**Figure 1:**
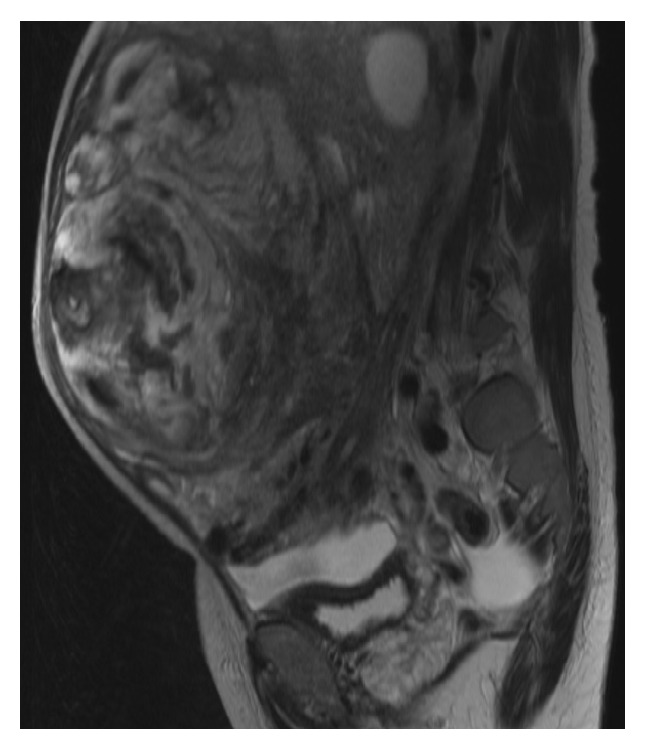
MRI image of a T2-weighted sagittal section. A huge abdominal tumor derived from the uterus, presenting with uneven intratumor signal.

**Figure 2:**
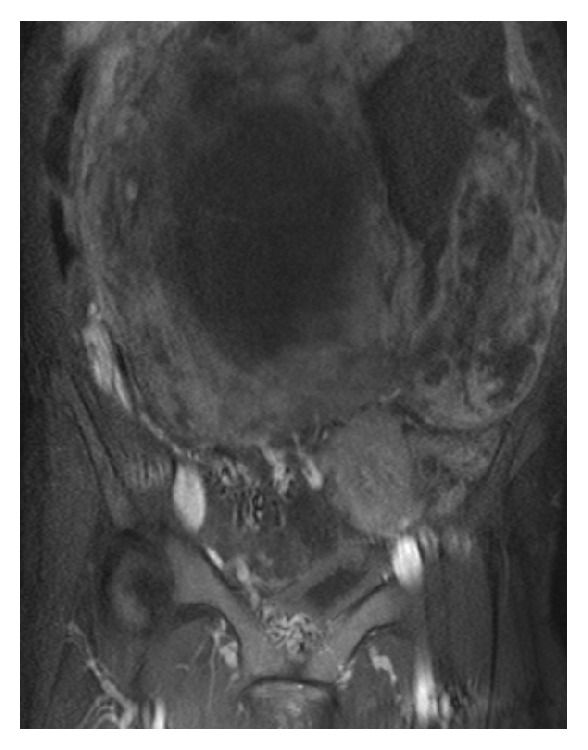
MRI image of a Gadolinium enhanced T1-weighted coronal section. The center of the abdominal tumor was not enhanced, implicating a suspicion of necrosis in the center.

A 48-year-old woman, gravida 1, para 1, visited the internal medicine department at another hospital with a complaint of abdominal fullness and weight loss of 10kg during the last year. A huge abdominal mass was palpated, and she was referred to the gynecology department to search for a tumor of uterine origin. She was premenopausal and had no significant past medical history. Physical findings revealed a large elastic hard mass extending from the xiphoid to the pubic bone. The magnetic resonance imaging (MRI) examination revealed a huge tumor on the uterine corpus, and a number of dilated vessels were observed between the tumor and the myometrium. Therefore, the tumor was suspected to derive from the uterus. The tumor showed an uneven signal on T2-weighted sagittal section (Figure 1), and the enhanced MRI study showed that the tumor edge but not the center was enhanced (Figure 2). As such, necrosis was suspected to have occurred in the center of the tumor. Uterine sarcoma was primarily suspected due to the large size, degeneration, and necrosis on MRI imaging. Computed tomography (CT) examination showed no lymph node swelling or distant metastasis. Preoperative laboratory testing revealed anemia (hemoglobin level, 5.6g/dl). We transfused 18 units of RCC before surgery. CT examination and ultrasonography on lower extremities indicated an absence of thrombosis. Preoperative serum levels of CEA, CA 19-9, CA 125, and LDH were within normal limits. A biopsy of the endometrium was not collected as the sounding examination of the endometrium was unsuccessful due to a deviated uterine cervix. At this point, preoperatively, we suspected the tumor was a leiomyosarcoma or leiomyoma with degeneration.

**Figure 3:**
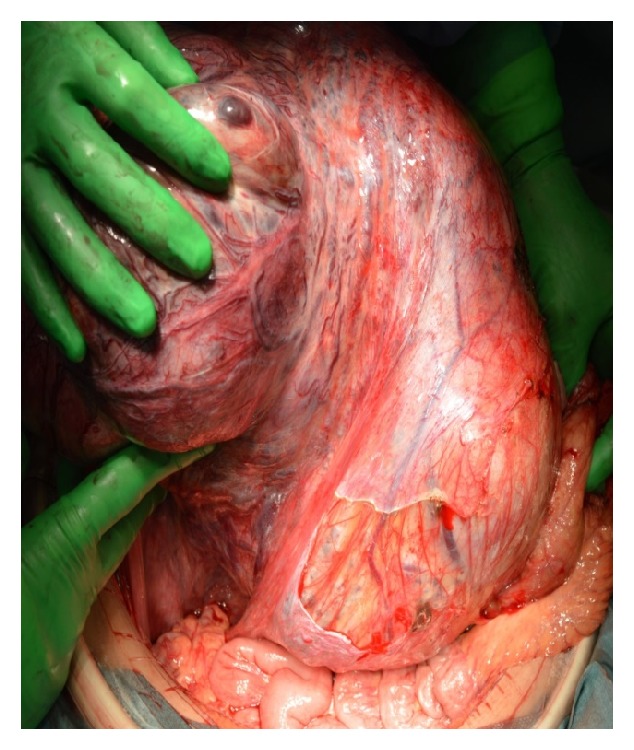
Intraoperative findings of the abdominal tumor. The surgery revealed a huge tumor occupying the space from the pelvis to the diaphragm. The tumor surface was smooth and hard with many dilated veins.

The patient underwent laparotomy, where we identified a huge tumor occupying a space from the pelvis to the diaphragm. The tumor surface was smooth and hard with many dilated veins (Figure 3). A massive tumor with a diameter of 30 cm was observed arising from the posterior uterine wall with a smooth contour and invaded the retroperitoneal cavity under the mesentery. The tumor was firmly adhered to both the mesentery and right ovary. There were no findings of extra-uterine dissemination. The intraoperative frozen section report for the uterine tumor was of degenerated myoma with no findings indicating malignancy. A total abdominal hysterectomy (TAH) and right salpingo-oophorectomy (RSO) were performed. The operation duration and blood loss were approximately 216 minutes and 1000 ml, respectively. The excised specimen weighed 7600 g.

**Figure 4:**
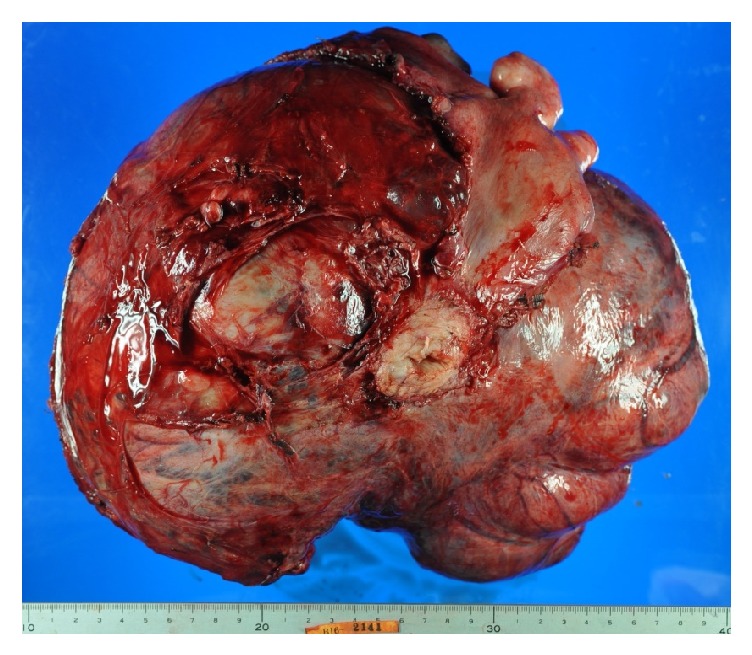
Gross findings of the excised tumor. The size of the tumor was 28 × 23 cm and the weight was 7600g. The tumor showed continuity with the posterior wall of the uterus.

**Figure 5:**
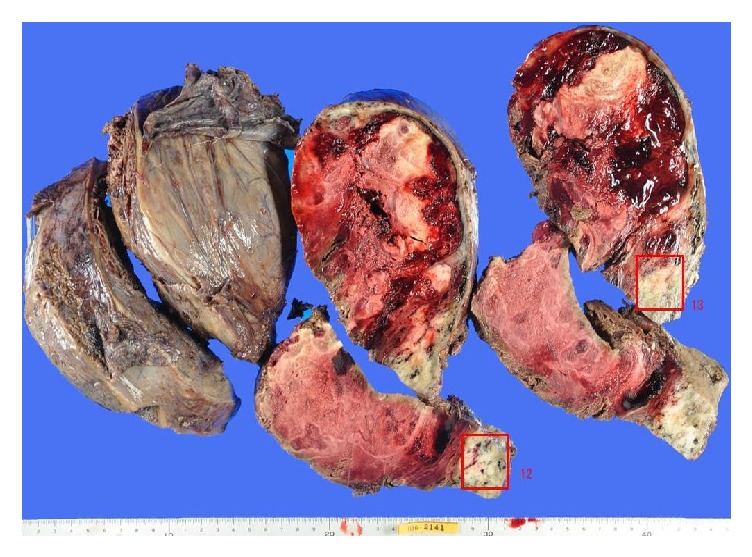
Macroscopic findings of sections of the tumor. On the sliced surface of the tumor, an obvious heterogeneous pattern was recognized within the mixture of a white homogeneous area, suggesting benign uterine fibroids, and a vulnerable part, due to bleeding and necrosis.

Macroscopic findings of the tumor revealed a well-circumscribed tumor showing extensive continuity with the posterior wall of the uterus, measuring 28 × 23 cm (Figure 4). On the sliced surface of the tumor, an obvious heterogeneous pattern was recognized within the mixture of a whitish homogeneous area, suggesting benign uterine fibroids, and a vulnerable area, due to bleeding and necrosis (Figure 5).

**Figure 6:**
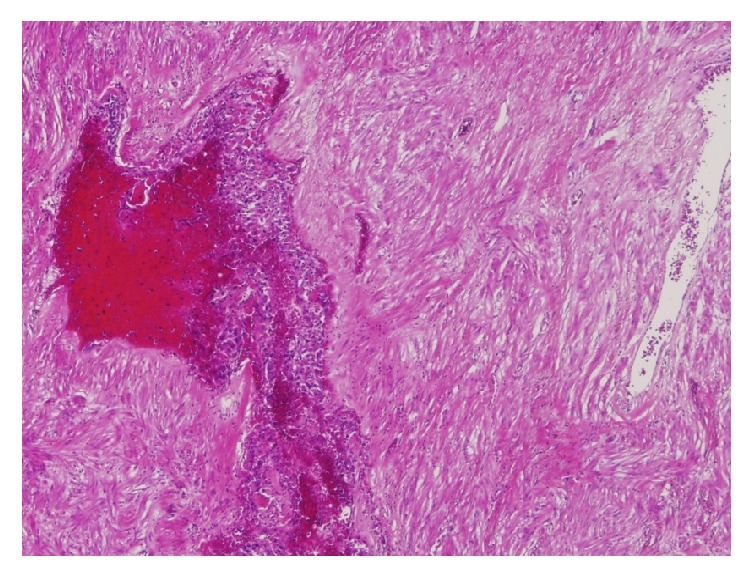
Microscopic findings of the tumor (H.E. stain; original magnification X40). The tumor was mostly composed of spindle-shaped cells, consistent with degenerated leiomyoma. Enlargement of blood vessels was observed within an area of about 2 cm × several mm, and proliferation of atypical cells showing a fine meshwork microvascular structure was observed in the blood vessel cavity.

**Figure 7:**
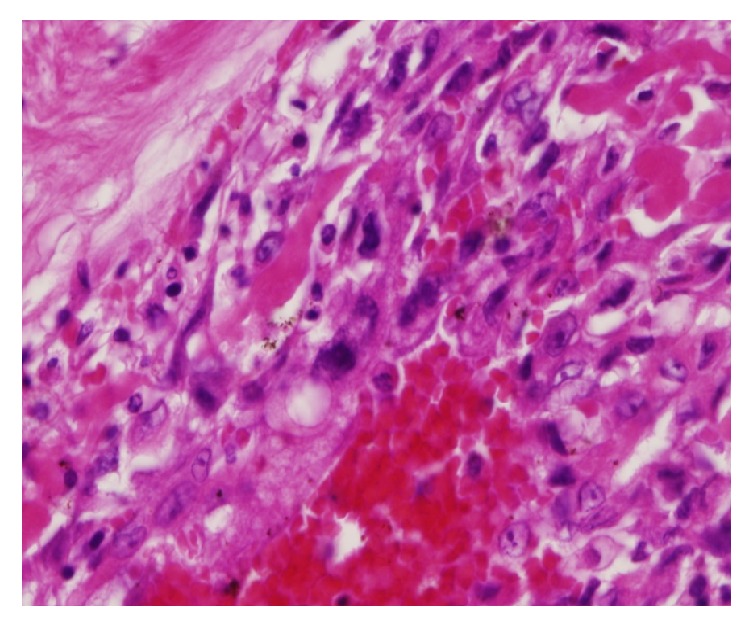
Microscopic findings of the tumor (H.E. stain; original magnification X400). These atypical cells consisted of various contours, such as cubic, polygonal, and short spindle shape. The nucleus was circular with a high degree of vacuolar enlargement and pleomorphism. Abnormal mitotic figures were also interspersed.

For the intraoperative frozen section, we examined three areas, namely, a white homogenous part, a necrotic part, and a cystic part, of which all were findings of a leiomyoma. In the permanent histological examination, 10 additional sections were collected from the tumor. The basic histological findings of all the sections were the same. The tumor was comprised of spindle-shaped cells, homologous to smooth muscle cells, which were arranged in bundles with areas of hyalinization, consistent with a degenerated leiomyoma. The tumor was mostly comprised of degenerated uterine leiomyoma. However, enlarged blood vessels were observed within an area of approximately 2 cm × several mm, and proliferation of atypical cells showing a fine meshwork microvascular structure was observed in the blood vessel cavity (Figure 6). These atypical cells consisted of various contours, such as cubic, polygonal, and short spindle shape. The nucleus was circular with a high degree of vacuolar enlargement and pleomorphism. Abnormal mitotic figures were also interspersed (Figure 7). A tumor derived from a blood vessel was thus considered, and malignancy was suggested by the presence of nuclear atypia and abnormal mitosis.

**Figure 8:**
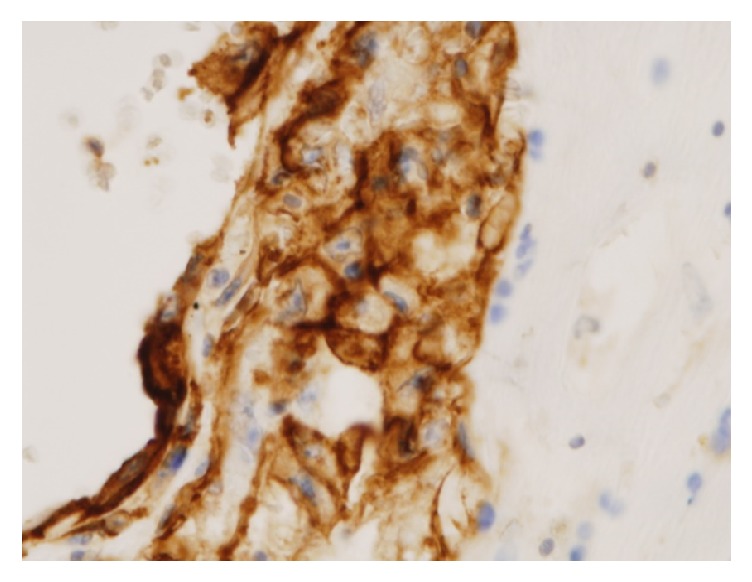
Immunohistochemical findings of the tumor (ERG; original magnification X400). The cytoplasm of the tumor cells was strongly positive for ERG stains.

**Figure 9:**
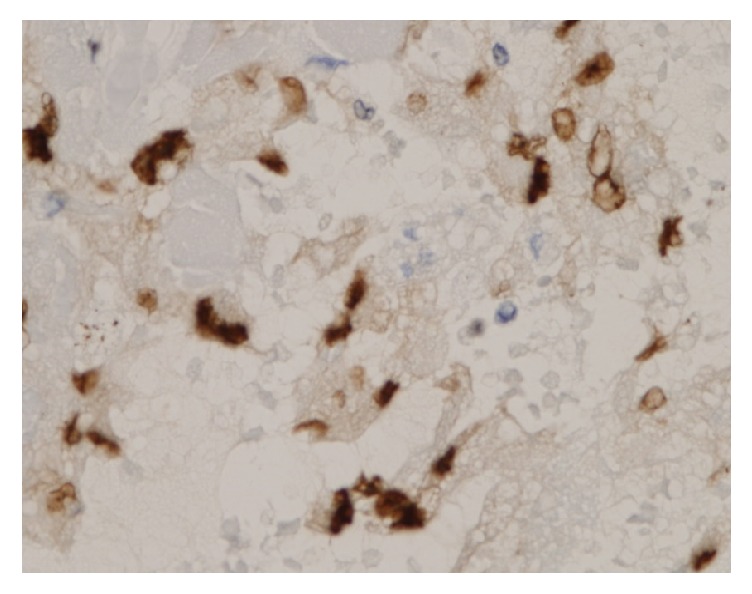
Immunohistochemical findings of the tumor (CD31; original magnification X400). The cytoplasm of the tumor cells was diffusely positive for CD31 stains.

Immunohistochemical analysis revealed the atypical tumor cells to be positive for ERG, CD31, and AE1/3 (Figures 8 and 9), partially positive for Factor VIII, and negative for *α*-SMA, desmin, H-caldesmon, EMA, CD34, and D2-40. From the above, the atypical tumor cells were of epithelial origin and the final diagnosis was epithelioid angiosarcoma arising in a degenerated uterine leiomyoma.

The efficacy of postoperative adjuvant therapy for angiosarcoma has not been demonstrated and there is currently no established chemotherapy regimen. In this case, because the atypical tumor was observed in the blood vessel cavity, we thought it could have been spread hematogenously throughout the body. Hence, we selected adjuvant chemotherapy rather than adjuvant radiotherapy. Six courses of combination adjuvant chemotherapy with paclitaxel (150mg/m2), epirubicin (50mg/m2), and carboplatin (area under the curve = 4) were administered in the present case, following referral to previous reported cases. No recurrence has been observed 10 months after the primary surgery.

## 3. Discussion

Soft tissue sarcoma accounts for less than 1 percent of all adult malignancies [4]. Less than 1% of all soft tissue sarcoma are angiosarcoma [2]. It can occur in any region of the body. Approximately half of the angiosarcomas occur in the skin, followed by breast and soft tissues. These account for 75% of angiosarcomas [5]. Chronic lymphedema and radiation are the most widely recognized predisposing factors for angiosarcoma of the skin and soft tissue. In general, angiosarcoma has an overall 5-year survival of approximately 35% [6]. Even with localized disease and optimal surgery conditions, only 60% of patients survive for more than 5 years. In advanced cases, the prognosis is poor with a median survival of 7 months [7].

Although angiosarcoma rarely occurs in the female genital tract [6], it has been reported to originate from the uterus, cervix, fallopian tube, ovary, uterine parametrium, broad ligament, and vagina [8]. To our knowledge, only 22 cases have been reported to date [8–25], as summarized in Table 1. Here we report the 23rd case in the literature.

Uterine angiosarcoma can occur in both premenopausal and postmenopausal women, although most women who develop uterine epithelioid angiosarcoma are postmenopausal. The most common symptom is vaginal bleeding, and in some cases patients come to the hospital with anemia or weight loss. The median age of the 23 patients was 61 years (range, 17-81 years). The uterus was characteristically large for almost all those women. It is clearly described with image inspection such as ultrasound, CT, and MRI. Our case also had a large uterus, but the patient was premenopausal. As such, this is considered a very rare case of angiosarcoma.

There are characteristic pathological findings, both grossly and microscopically. Grossly, the tumor is composed of whitish or grayish hemorrhagic tissue with areas of necrosis or calcification. It may have a lobulated pattern [10, 11, 13]. Our case had similar macroscopic findings and angiosarcoma was detected from whitish hemorrhagic tissue.

The histological features can vary and as such, distinguishing angiosarcoma from a benign proliferative or inflammatory lesion with light microscopy is often difficult. Microscopically, the tumors contain irregular rudimentary vascular significant pleomorphism and nuclear hyperchromatism, with frequent mitotic figures. In addition, there are numerous solid areas composed of cells that have eosinophilic cytoplasm with occasional vacuolization and round nuclei. Binucleated and multinucleated giant tumor cells are also present [11, 15].

Angiosarcomas are typically positive for endothelial markers including CD31, CD34, and Factor VIII. Muscle markers such as actin, desmin, and S-100 protein are usually negative. Concerning the epithelial marker AE 1/3, such tumors are often negative but were positive in the present case [15]. Immunohistochemical analysis is therefore important in confirming the diagnosis.

There is a lack of consensus on the optimal treatment and factors influencing the prognosis for angiosarcoma of the uterus. Most published reports of angiosarcoma treatment are retrospective case series. Wide resections are often required because of the invasive and multifocal nature of angiosarcoma.

In the past 50 years, all patients were initially treated by TAH with bilateral salpingo-oophorectomy (BSO). The limited information available in the literature does not support a routine pelvic and/or para-aortic lymphadenectomy.

Some patients who underwent postoperative adjuvant chemotherapy and radiotherapy have survived for more than 4 years but are limited. Chemotherapy is also performed in various combinations, but there is no established regimen. Paclitaxel is currently the most commonly used drug for angiosarcoma [26]. In one report, eight of nine patients with scalp angiosarcoma experienced a major response, four with partial responses and four clinically complete responses with paclitaxel [7]. However, the largest study of adjuvant chemotherapy in soft tissue sarcoma has failed to demonstrate any survival advantage [27]. High dose adjuvant radiotherapy (>50 Gy) and wide treatment field are recommended due to the high risk of local recurrence. No formal radiotherapy trials have been done, but retrospective studies suggest that it improves local control and overall survival [28]. There is no compelling evidence for adjuvant chemotherapy and radiotherapy. We administered combined chemotherapy, including paclitaxel as adjuvant chemotherapy, with reference to previous case reports

Uterine angiosarcoma often recurs within a few months, and its prognosis is very poor (Table 1). However, there are 4 cases with no recurrence for more than 3 years following surgical treatment alone. In three cases, the lesion was as small as 5 cm or less, and invasion of the uterine myometrium was less than half [13, 17, 23]. Based on these findings, if the tumor diameter is 5 cm or less with less than half of the myometrium being invaded, it may be possible to extend the prognosis with only wide resection surgery.

Among the 23 cases, there are only six cases of epithelioid angiosarcoma arising in the leiomyoma of the uterus. It suggests that the increased vascular proliferation secondary to a mechanical pressure effect of adjacent leiomyomas might have induced the endothelial neoplastic transformation [16]. However, most uterine angiosarcomas were not associated with uterine leiomyoma. This finding suggests that whereas uterine angiosarcoma can arise in association with uterine leiomyomas, it more commonly develops de novo [21].

Recently, laparoscopic treatment has been widely conducted in the gynecological field. Petrillo et al. reported cases of low grade endometrial stromal sarcoma occurred in the site of trocar placement five years after laparoscopic myomectomy with intraabdominal morcellation. Therefore, we propose that laparoscopic procedure should be avoided when treating leiomyoma with potential malignant tumors [29].

Suzuki et al. identified breakages at three loci, i.e., YWHAE (17p13), FAM22A (10q23), and FAM22B (10q22) in the case of uterine angiosarcoma. These findings suggest that an abnormality in the loci of YWHAE, FAM22A, and FAM22B may contribute to the development of uterine angiosarcoma [23].

We described a patient with a primary angiosarcoma arising from a leiomyoma that was treated initially by surgery and adjuvant chemotherapy. If the tumor diameter is 5 cm or less with less than half of the muscle layer being infiltrated, it may be possible to extend the prognosis by performing wide resection it. Moreover, in the presence of a huge uterus, angiosarcoma may be contained within. Consequently, detailed histological diagnosis for whitish or grayish hemorrhagic tissue with areas of necrosis or calcification is required.

## Figures and Tables

**Table 1:** **Summary of the uterine epithelioid angiosarcomas in literature review**. TAH: total abdominal hysterectomy, BSO: bilateral salpingo-oophorectomy, LSO: left salpingo-oophorectomy, RSO: right salpingo-oophorectomy, POM: partial omentectomy, PLN: pelvic lymphadenectomy, PAN: para-aortic lymphadenectomy, NED: no evidence of disease, DOD: dead of disease.

Case No.	Author (Year)	Age	size	Presentation	Treatment	Outcome (months)
1	Purola and Strandell (1967)	57	−	Vaginal bleeding	TAH, BSO appendectomy, RT	NED, 18mo

2	Ehrmann and Griffiths (1979)	17	9	Vaginal bleeding	TAH, LSO, CT, RT	DOD, 84mo

3	Ongkasuwan (1982)	70	11	Malaise weight loss	TAH, BSO	NED, 5mo

4	Witkin (1987)	71	15×17×7	Vaginal bleeding	TAH, BSO, RT	Reccurence 6mo

5	Milne (1990)	76	18	Vaginal bleeding urinary retention	TAH, BSO	DOD, 6mo

6	Quinonez (1991)	65	8	Vaginal bleeding	TAH, BSO, PLN CT, RT	NED, 48mo

7	Lack (1991)	71	−	Vaginal bleeding	TAH, BSO, RT	DOD, 2mo

8	Tallini (1993)	56	30×24	Vaginal bleeding	TAH, BSO, POM PAN, appendectomy,	DOD, 7mo

9	Drachenberg (1994)	58	12	Vaginal bleeding	TAH, BSO, CT, RT	DOD, 2mo

10	Schammel (1998)	49	29×29×19	Vaginal mass	TAH, BSO	DOD, 3mo

11	Schammel (1998)	58	12	Vaginal bleeding	TAH, BSO, CT, RT	DOD, 2mo

12	Schammel (1998)	70	5×3×3	Vaginal bleeding	TAH, BSO	NED, 37mo

13	Schammel (1998)	75	6×6×5	Vaginal bleeding	TAH, BSO	DOD, 7mo

14	Mendez (1999)	59	12-wk-size uterus	Vaginal bleeding	TAH, BSO	DOD, 2.5mo

15	Konishi (2007)	62	17×15×7.5	Anemia	TAH, BSO, CT	NED, 2mo

16	Cardinale (2008)	81	8×7×5	Lower abdominal pain anemia	TAH, BSO	DOD, 6mo

17	Cardinale (2008)	35	25	Shortness of breath dry cough	TAH, BSO	No information

18	Olawaiye (2008)	54	11×6	Enlarged uterus	TAH, BSO	NED,12mo

19	Hwang and Lim (2013)	61	12×10×9	Vaginal bleeding	TAH, BSO, PAN, RT	No information

20	Suzuki (2014)	64	7.5×5.5×3.5	Vaginal bleeding	TAH, BSO	DOD, 50mo

21	Yankun Liu (2015)	56	11×8×7	Anemia	TAH, BSO	No information

22	Strickland (2017)	67	21×18×15	Fatigue, weight loss	TAH, BSO	DOD, 2mo
